# Manometric Evaluation of Esophageal Motility in Patients With Non-cardiac Chest Pain: A Retrospective Study From a Tertiary Center in Abu Dhabi, United Arab Emirates

**DOI:** 10.7759/cureus.98290

**Published:** 2025-12-02

**Authors:** Mathew Vadukoot Lazar, Amit Hanmant Shejal, Hadik Patel, Sanil Kumar Raju, Jessymol Joseph, Divya Jose, Baiju Faizal Puthenkote, Monica Jadhav, Sarath Babu, Rakesh Gupta

**Affiliations:** 1 Gastroenterology and Hepatology, Lifecare Hospital, Musaffah, Abu Dhabi, ARE; 2 Internal Medicine, Lifecare Hospital, Musaffah, Abu Dhabi, ARE; 3 Research and Health Innovation, Lifecare Hospital, Musaffah, Abu Dhabi, ARE; 4 Cardiology, Lifecare Hospital, Musaffah, Abu Dhabi, ARE; 5 Pulmonology, Lifecare Hospital, Musaffah, Abu Dhabi, ARE

**Keywords:** achalasia, barium swallow study, dysphagia, esophageal motility, gi endoscopy, hiatus hernia, high-resolution manometry, non-cardiac chest pain, united arab emirates, vomiting

## Abstract

Non-cardiac chest pain (NCCP) is a frequent clinical problem that often mandates intensive investigations for the exclusion of cardiac etiology. Esophageal motility disorders are a leading cause of NCCP; however, data from the Middle East are lacking. This was a retrospective study of 41 male patients (n=41; 100%) aged 23-57 years, who were referred to a tertiary care institution in Abu Dhabi, United Arab Emirates, with NCCP. All patients had high-resolution manometry (HRM), upper gastrointestinal endoscopy, and laboratory testing. HRM showed ineffective esophageal motility (IEM) as the most common abnormality in 12 patients (n=12; 30%), followed by distal esophageal spasm (DES) in five patients (n=5; 12.5%), while achalasia and esophagogastric junction (EGJ) outflow obstruction were infrequent. Importantly, 18 patients (n=18; 45%) with routine endoscopy had abnormal HRM patterns, reflecting the shortcomings of endoscopy alone. We did not find a significant correlation between endoscopic and HRM findings. These findings support the importance of including HRM in NCCP diagnostic algorithms to enhance accuracy and inform personalized, patient-centered management in this area.

## Introduction

Non-cardiac chest pain (NCCP) is a symptom of widespread presentation and a significant diagnostic dilemma for all clinicians globally. Recurrent attacks of chest pain characterize it as in angina pectoris, except that such attacks are triggered without any apparent cardiovascular disease [1]. NCCP is a significant proportion of emergency department and outpatient clinic presentations and leads to excessive health resource use. To exclude the possibility of ischemic heart disease, patients are frequently referred to undergo comprehensive cardiac testing such as an electrocardiogram, stress testing, and even angiography. After eliminating cardiac etiologies, clinicians are then left with the challenging issue of identifying other etiologies, most of which involve the gastrointestinal tract [2]. Among these, gastroesophageal reflux disease and esophageal motility disorders are of relevance, which account for almost 50-60% of patients with NCCP [3]. The similarity of symptomatology between cardiac and non-cardiac causes not only complicates the diagnosis but also delays adequate management, resulting in patient anxiety and repeated hospital visits.

High-resolution manometry (HRM) has become the gold standard diagnostic tool for evaluating esophageal motility disorders. Unlike conventional manometry, HRM enables the spatiotemporal mapping of esophageal pressure dynamics, which can be used to classify motility patterns using standardized criteria such as the Chicago Classification [4]. With HRM, clinicians can distinguish among different esophageal motility disorders, including ineffective esophageal motility (IEM), distal esophageal spasm (DES), achalasia, and esophagogastric junction (EGJ) outflow obstruction. These observations are of vital importance for personalizing therapeutic approaches in patients, from dietary adaptations and pharmacotherapy to endoscopic or surgical techniques. Despite its diagnostic importance, HRM is underutilized in many regions, particularly in developing healthcare facilities where access to specialized gastroenterology services may be limited [5].

In the Middle East, and the United Arab Emirates in particular, studies related to NCCP and its esophageal etiology are limited. However, most of the literature on esophageal motility disorders originates from Western countries or South Asia, and a considerable gap remains in understanding regional patterns [6,7]. Several studies have shown that the prevalence and manifestation of motility disorders can be affected by geographic, genetic, environmental, and lifestyle factors [8]. For instance, estimates from Western cohorts indicate an IEM prevalence in the range of 20-25%, while Indian studies suggest a modestly higher prevalence of approximately 32%. Environmental factors, such as dietary intake (e.g., high intake of fizzy drinks) and vitamin D deficiency, which are common in the UAE population, may also contribute to differences in esophageal motility [9,10]. One of the problems for clinicians is the lack of local population information and the fact that diagnostic criteria and treatment guidelines developed for other populations may not be entirely relevant for the clinicians' local patients.

In view of these gaps, there is a crucial need to perform region-specific studies to establish baseline prevalence, diagnostic correlation, and implications for treatment of NCCP patients in the United Arab Emirates. We hypothesized that HRM would identify a high prevalence of esophageal motility abnormalities which are undetected by endoscopy among patients with NCCP. The primary objective was to determine the prevalence and distribution of esophageal motility disorders according to the Chicago Classification v3.0 [11]. The secondary objectives were (1) to correlate HRM findings with endoscopic results, specifically assessing the frequency of abnormal motility in patients with normal endoscopy, and (2) to identify any associations between manometric abnormalities and laboratory parameters or specific symptoms like dysphagia.

## Materials and methods

Study design and setting

This study was conducted retrospectively as a cohort study at Lifecare Hospital, Musaffah, a tertiary care center in Abu Dhabi, United Arab Emirates. The study was conducted for patients with NCCP who had undergone a complete evaluation to rule out cardiovascular causes. This setting was selected because the hospital is a referral center for advanced gastroenterological diagnostics, including HRM and endoscopic evaluation. The study was conducted over a one-year period, from November 2017 to December 2018, and all consecutive patients who met the inclusion criteria were included in the study. The retrospective nature made available existing electronic medical records and allowed all relevant diagnostic data to be systematically reviewed without direct patient contact. Ethical approval was obtained from the Burjeel Holdings Institutional Review Board (IRB) under approval number BH/REC/105/25. Since the study was retrospective in nature, a waiver of informed consent was obtained in accordance with institutional policy for archival studies. Strict data protection and confidentiality were ensured, and all identifiable patient information was anonymized before analysis.

Participants and eligibility criteria

The study population consisted of 41 male patients aged from 23 to 57 years, which is representative of the demographic distribution of patients presenting to the hospital during the study period. All patients had their primary complaint as NCCP and had undergone a full cardiac workup. Cardiac etiology was definitively ruled out based on the following criteria: (1) a normal 12-lead electrocardiogram (ECG), (2) normal serial cardiac enzymes (troponin I/T) measured at presentation and 4-6 hours later, and (3) a regular functional cardiac test (either an exercise stress test or dobutamine stress echocardiography) or a routine coronary CT angiography, all performed within six months of presentation. Only patients fulfilling all these negative cardiac criteria were eligible for inclusion. Symptoms of dysphagia and recurrent vomiting were defined by their documented presence in the clinical history within the patient's electronic medical record. The inclusion criteria were adult male patients aged 18 years or older, with clinical features suggestive of NCCP, and both HRM and upper gastrointestinal endoscopy completed as part of the diagnostic workup. Specifically, the study comprised males, as, in this local area, the patient population referred to and seen at a gastroenterology service is predominantly male due to health-seeking and referral patterns. Exclusion criteria included patients with a previous history of esophageal or gastric surgery, known systemic connective tissue disease (such as scleroderma), incomplete or absent manometry or endoscopy results, and coexisting neurological or psychiatric conditions that would confound symptom interpretation. These criteria provided a homogeneous study sample, which allowed for a more direct understanding of HRM and endoscopic findings.

Diagnostic procedures 

All participants received a standardized diagnostic workup, including HRM, upper endoscopy, and laboratory investigations.

HRM

HRM was performed using a Solar GI HRM system (Laborie, Portsmouth, New Hampshire, United States), equipped with a 36-channel solid-state catheter featuring circumferential pressure sensors spaced 1 cm apart. The protocol adhered to the international standards outlined in the European Society of Neurogastroenterology and Motility (ESNM) recommendations [4]. Patients were required to fast for a minimum of six hours prior to the procedure. The catheter was trans-nasally placed and positioned to capture pressure from the pharynx to the stomach. The manometry study included a baseline period and a 10-swallow protocol wherein each participant ingested 10 5 ml aliquots of room-temperature water in the supine position.

Manometric data were analyzed using the Solar GI HRM software. The following key diagnostic thresholds from the Chicago Classification v3.0 [11] were applied: IEM: ≥50% of ineffective swallows with a distal contractile integral (DCI) <450 mmHg·cm·s; DES: ≥20% of swallows with a normal integrated relaxation pressure (IRP) and premature contraction (distal latency (DL) <4.5 seconds); achalasia: elevated median IRP (>15 mmHg) and 100% failed peristalsis (DCI <100 mmHg·cm·s); and EGJ outflow obstruction: elevated median IRP (>15 mmHg), preserved peristalsis, and not meeting the criteria for achalasia. All HRM studies were interpreted by two experienced gastroenterologists, with any diagnostic discrepancies resolved by consensus.

Upper Endoscopy

Gastroenterologists with high-definition video endoscopes performed upper gastrointestinal endoscopy. The clinical results were documented and were evaluated with the structural and mucosal alterations, namely, hiatus hernia, pangastritis, duodenitis, erosive gastritis, and esophagitis. Biopsies were performed where clinically felt necessary. The definition of hiatus hernia was the axial movement of the gastroesophageal junction with more than 2 cm above the hiatus diaphragm. Gastritis was graded based on the Sydney System, and esophagitis was graded based on the Los Angeles Classification.

Laboratory Investigations

All patients had baseline laboratory testing, including complete blood count (CBC), eosinophil percentage, and C-reactive protein (CRP). Stomach *Helicobacter pylori *(*H. pylori*) status was established with stool antigen testing and recorded as a negative or positive result. Imaging studies included barium swallow for functional testing and an abdominal ultrasound to detect fatty liver changes or other hepatobiliary abnormalities.

Data collection and operational definitions

Data were reviewed from electronic medical records using a standardized collection form to ensure homogeneity. The form captured demographics, symptom profiles (chest pain, dysphagia, regurgitation, vomiting), laboratory data, endoscopic findings, and HRM results. Symptom documentation followed structured templates used routinely in the hospital's electronic medical records. Data abstraction was performed by two investigators independently, and discrepancies were resolved by cross-checking with the original clinical records.

Data analysis

Statistical analyses were conducted using IBM SPSS Statistics for Windows, Version 26.0 (Released 2019; IBM Corp., Armonk, New York, United States). This study utilized a convenience sample, including all consecutive patients who met the inclusion criteria during the one-year study period (November 2017 to December 2018). No formal sample size calculation was performed a priori. A complete-case analysis was employed; no data imputation was performed for missing variables, and patients with missing HRM or endoscopy data were excluded. Continuous variables (age, CBC, CRP, eosinophil percentage) were presented as mean plus standard deviation (SD). Categorical variables, such as the presence or absence of particular endoscopic or manometric findings, were reported as frequencies and percentages. Descriptive statistics were used initially to describe the demographic and clinical characteristics of the study population. This was for age distribution, prevalence of symptoms, and frequency of several endoscopic and manometric diagnoses. Chi-squared tests were performed to assess the associations between categorical variables such as the relation between HRM abnormalities and endoscopic findings (e.g., hiatus hernia vs. IEM) for inferential analysis. Logistic regression analysis was performed to find independent predictors of abnormal HRM using age, CBC, CRP, and eosinophil levels as potential predictors. Results were expressed as odds ratios (OR) with 95% confidence intervals (CI). P<0.05 was taken as statistically significant. Results were visualized in bar charts, heatmaps, and other graphical displays to show symptom distribution, endoscopic findings, and HRM outcomes. This was done for age group distributions, symptom prevalence, and the relation between HRM and endoscopic findings.

## Results

Demographics and symptoms

**Figure 1:**
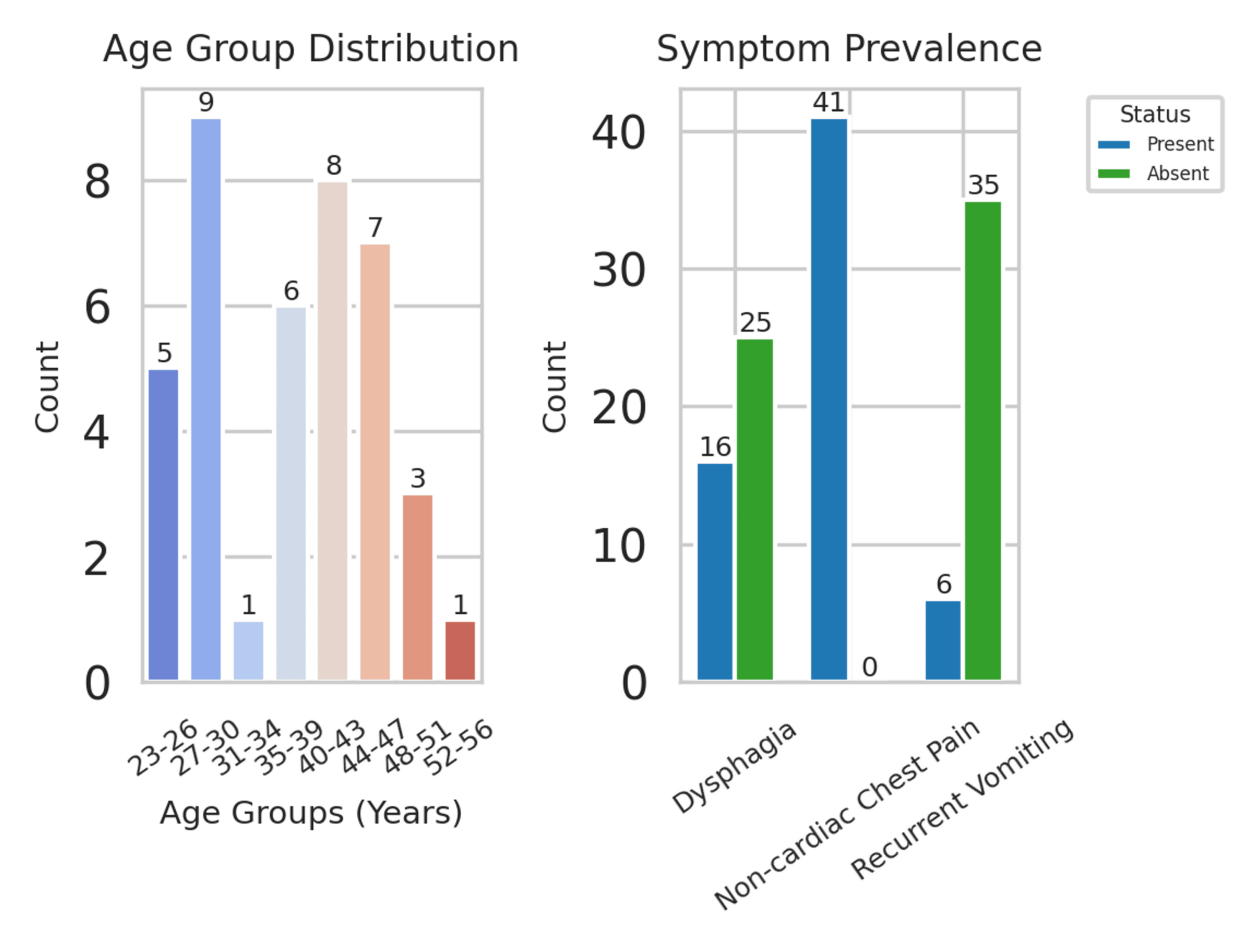
Demographics and symptoms

A total of 41 male patients (n=41; 100%) presenting with NCCP were included in this study. Participants ranged in age from 23 to 57 years (n=41; 100%), with an average age of approximately 39 years. The patients were stratified into eight different age groups to facilitate a better understanding of the distribution of NCCP cases across the stages of adulthood. More than half of the patients fell into the age groups of 27-30 and 40-43 years, comprising the majority of the total sample. The lowest incidence was observed in the oldest age group (52-56 years), indicating that NCCP and related esophageal disorders were more prevalent in younger and middle-aged adults in this group (Figure 1).

Symptom prevalence was determined according to three major criteria: dysphagia, NCCP (this was present in all patients as one of the primary inclusion criteria), and recurrent vomiting. Dysphagia was noted in 16 patients (n=16; 40%), and recurrent vomiting was noted in six patients (n=6; 15%). NCCP (the defining symptom of the study) was consistently reported across the cohort. Dysphagia was a common secondary symptom associated with manometric abnormalities, including IEM. This raises the possibility that dysphagia is an early clinical sign of undiagnosed esophageal motility disorders in NCCP patients.

Endoscopic findings

**Figure 2:**
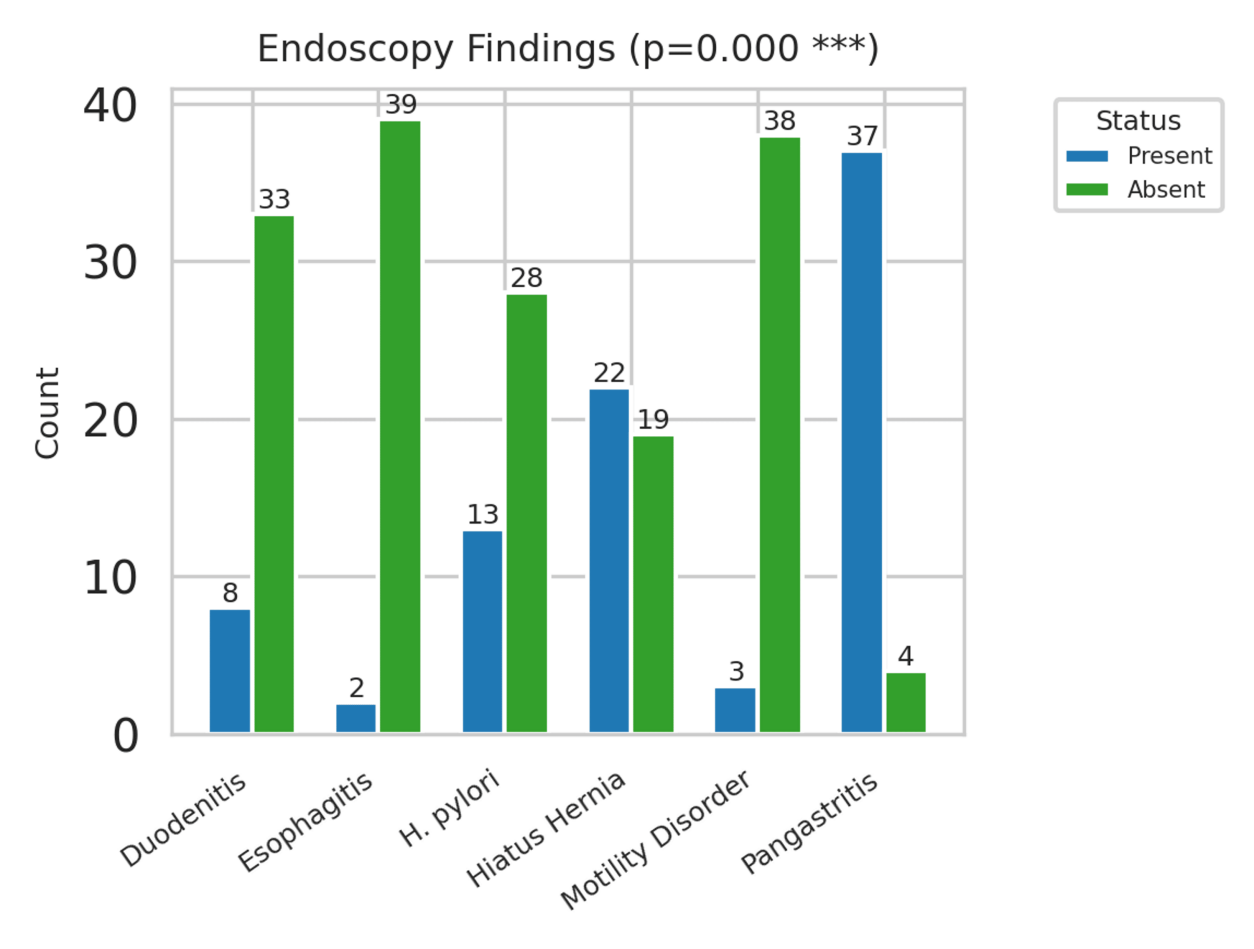
Endoscopic findings' distribution

Upper gastrointestinal endoscopy revealed a range of abnormalities among the study group. Pangastritis was the most frequent finding observed in 37 patients representing 92.5% of the cohort (n=37; 92.5%), indicating a high frequency of inflammatory changes in the gastric mucosa. Hiatus hernia was found in 22 patients (n=22; 55%), suggesting a potential relationship with NCCP symptoms. Duodenitis was present in eight patients (n=8; 20%), while esophagitis was relatively rare, being found only in two patients (n=2; 5%). *Helicobacter pylori *(*H. pylori*) positivity was found in 13 patients (n=13; 32.5%), consistent with regional prevalence data for the United Arab Emirates, with the highest positive rate observed in pangastritis and duodenitis, suggesting that *H. pylori* serves as an important factor in the inflammatory processes of the upper gastrointestinal mucosa. Most endoscopic abnormalities consisted of inflammatory rather than structural lesions, consistent with the hypothesis that NCCP in this cohort is usually functional rather than obstructive disease. Of particular interest, 18 patients (n=18; 45%) had completely normal endoscopic findings, suggesting that endoscopy alone may be inadequate for attributing NCCP etiology (Figure 2).

HRM findings

**Figure 3:**
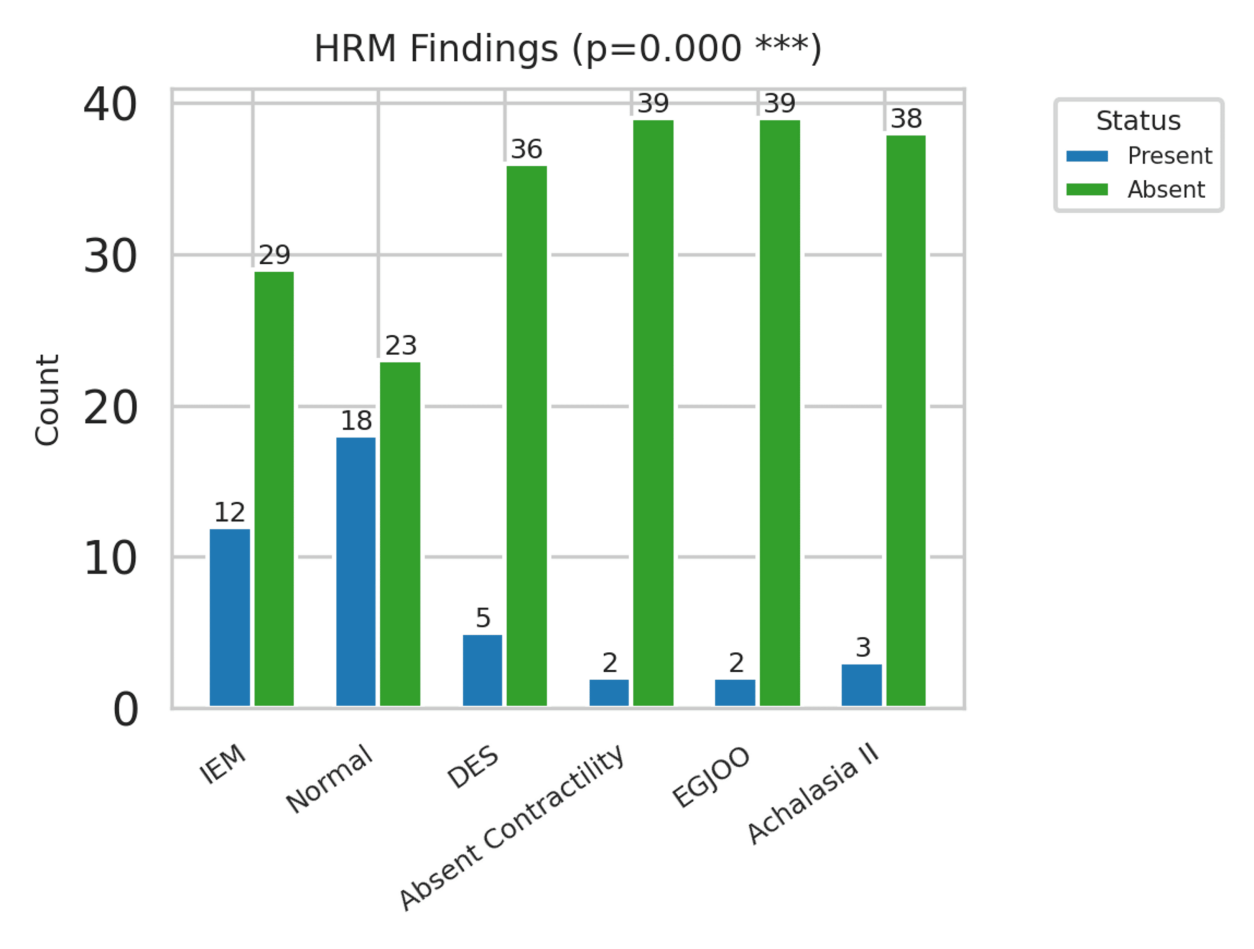
Manometric findings' distribution HRM: high-resolution manometry; IEM: ineffective esophageal motility; DES: distal esophageal spasm; EGJOO: esophagogastric junction outflow obstruction

HRM provided important insights into NCCP esophageal motor physiology. According to the Chicago Classification v3.0, normal esophageal motility was reported in 18 patients (n=18; 45%), suggesting that almost half of the cohort had no motility abnormalities even though they presented with NCCP. Of the abnormal HRM findings, IEM was the most common diagnosis, occurring in 12 patients (n=12; 30%). This was followed by DES in five patients (n=5; 12.5%). Less frequent findings were type II achalasia in three patients (n=3; 7.5%) and EGJ outflow obstruction in two patients (n=2; 5%). Type I or type III achalasia was not found. This distribution indicates that IEM is the major motility disorder in patients with NCCP in this regional setting, similar to the results from the international literature. Given the relatively low prevalence of achalasia and EGJ outflow obstruction, it is important to distinguish functional motility disorders from structural or obstructive etiologies (Figure 3).

Comparison between endoscopic and manometric findings

**Figure 4:**
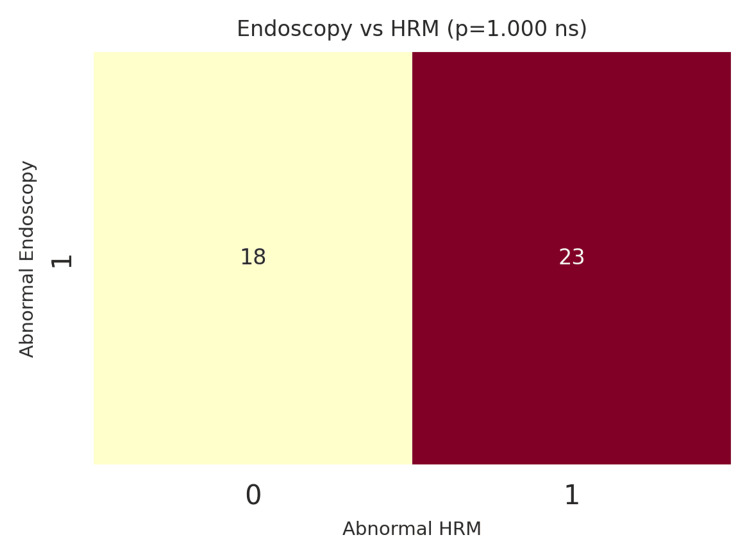
Normal vs. abnormal: endoscopy vs. HRM HRM: high-resolution manometry

Therefore, one of the purposes of this study was to investigate the correlation between endoscopic and manometric findings. Of the 41 patients (n=41; 100%), 18 (n=18; 45%) had normal endoscopy but abnormal HRM, suggesting that a significant proportion of cases of NCCP may have underlying motility disorders that cannot be detected using endoscopy alone. In contrast, 23 patients (n=23; 57.5%) showed abnormalities on both endoscopy and HRM. This discordance emphasizes the complementary nature of endoscopy and HRM for the diagnosis of NCCP. A chi-squared test was performed to statistically evaluate the association between endoscopic and HRM abnormalities, yielding a chi-squared value of 0.0 with a degree of freedom (df) of 1, an effect size (Cramer's V) of 0.0, and a p-value of 1.0. This indicates that endoscopic findings may not be a good predictor of motility pathophysiology, further supporting the role of both tests in the clinical assessment of NCCP patients (Figure 4).

**Figure 5:**
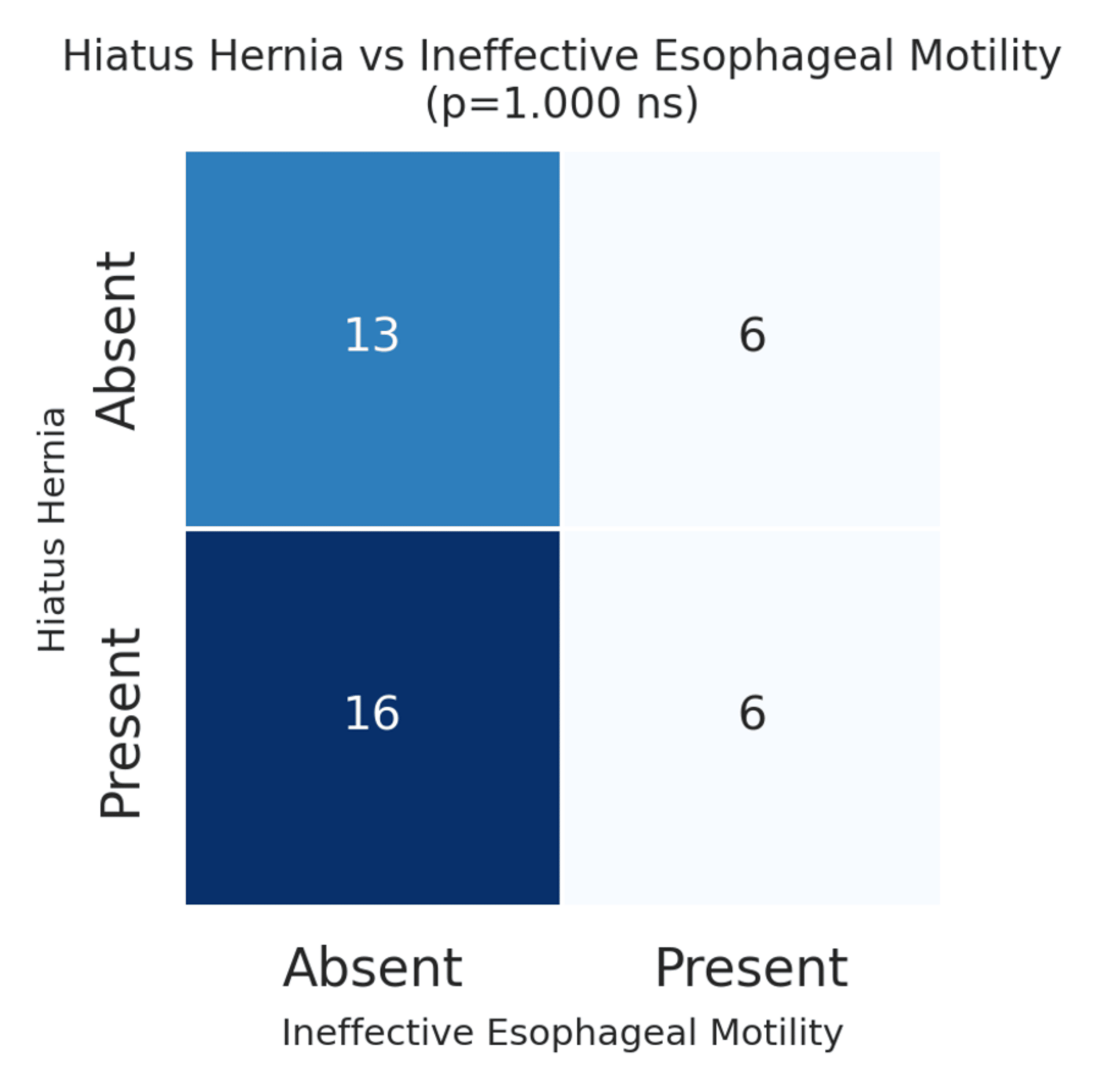
Hiatus hernia vs. ineffective esophageal motility

A secondary analysis explored the relationship between specific endoscopic findings and motility disorders. For example, the association between hiatus hernia and IEM was examined, as both conditions involve the EGJ. Of the 22 patients (n=22; 55%) with hiatus hernia, six also had IEM. However, statistical testing revealed no significant association between these two variables (p>0.05). This indicates that while hiatus hernia and IEM may co-occur, one does not necessarily predict the presence of the other (p=1.000) (Figure 5).

Logistic regression analysis

To identify independent predictors of abnormal HRM findings, a binary logistic regression model was constructed using age, CBC, eosinophil percentage, and CRP levels as potential predictors. The dependent variable was the presence or absence of abnormal HRM results. The model showed that none of the included variables were statistically significant predictors of abnormal HRM, as indicated by their respective p-values. Age (p=0.705; OR=0.99; 95% CI: 0.91-1.06), CBC (p=0.162; OR=0.74; 95% CI: 0.48-1.13), eosinophils (p=0.493; OR=1.06; 95% CI: 0.89-1.26), and CRP (p=0.175; OR=1.08; 95% CI: 0.96-1.22) all failed to reach significance. The overall model fit was modest, with a pseudo-R-squared value of 0.084, suggesting that other unmeasured factors may play a more important role in determining motility abnormalities in NCCP patients. The regression table in APA style is presented in Table 1.

**Table 1:** Logistic regression predicting abnormal HRM findings (n=41) SE: standard error; Wald χ²: Wald chi-square; OR: odds ratio; CI: confidence interval; CBC: complete blood count; CRP: C-reactive protein; HRM: high-resolution manometry

Predictor	B	SE	Wald χ²	p	OR (95% CI)
Constant	4.63	3.88	1.19	0.233	-
Age (years)	-0.01	0.04	0.14	0.705	0.99 (0.91, 1.06)
CBC	-0.31	0.22	1.95	0.162	0.74 (0.48, 1.13)
Eosinophils	0.06	0.09	0.47	0.493	1.06 (0.89, 1.26)
CRP	0.08	0.06	1.84	0.175	1.08 (0.96, 1.22)

The lack of significance of these clinical and laboratory variables indicates that mechanisms in addition to routine blood work and demographic variables play an important role in determining abnormal motility patterns. This reinforces the need for specialized diagnostic tools for NCCP, such as HRM.

## Discussion

Key findings and interpretation

This paper presents an in-depth analysis of esophageal motility and endoscopic observations in patients presenting with NCCP at a tertiary care hospital in Abu Dhabi, United Arab Emirates. The findings support the multifaceted and complex nature of NCCP, emphasizing the importance of employing supplementary diagnostic modalities to ensure an accurate diagnosis of the underlying pathology. The most striking finding was IEM, which accounted for the most frequent manometric defect in 30% of the research population. This finding is consistent with the general literature, which reports that IEM is the most frequent abnormal motility disorder observed in patients with NCCP. IEM represents fragmented or weak peristalsis, which can lead to inadequate esophageal clearance. This stasis can cause non-acidic regurgitation, distension, and chest pain, even in the absence of severe gastroesophageal reflux disease. This finding suggests that IEM represents a principal contributor to NCCP. The relatively small EGJ numbers of DES, type II achalasia, and outflow obstruction emphasize the heterogeneity of esophageal motility disorders and the necessity of comprehensive functional assessment to prevent misdiagnosis or missed diagnoses.

Another important finding was the mismatch between the morphological findings on endoscopy and the functional abnormalities detected by HRM. Whereas pangastritis and hiatus hernia were the most frequent endoscopic findings, almost half of the patients with normal endoscopic findings were found to have abnormal HRM patterns. This emphasizes the inadequacy of endoscopy alone in the diagnostic workup of NCCP. The absence of a statistically significant correlation further supports the complementary role of multimodal diagnostics. Notably, simple demographic and laboratory data, such as age, CBC, eosinophils, and CRP, did not prove to be good predictors of abnormal HRM outcomes in logistic regression analysis. This implies that special diagnostic tests are yet to be eliminated and cannot be replaced by clinical or laboratory tests in a routine manner.

Our cohort consists of 18 patients (n=18; 45%) with normal esophageal motility on HRM. It is a significant and clinically valuable result. A normal manometry study effectively rules out major motility disorders such as achalasia or DES. This narrows the differential diagnosis, preventing unnecessary invasive treatments. In this substantial subset of patients, the symptoms are likely related to functional heartburn, which is characterized by visceral hypersensitivity in which normal reflux events or esophageal contractions are perceived as painful, or non-erosive reflux disease (NERD), which can be missed by endoscopy and requires pH-impedance monitoring for confirmation. We should also consider the influence of central sensitization, often mediated by psychosocial comorbidities, which can profoundly alter esophageal sensory processing. A normal HRM outcome, therefore, is not a diagnostic end but a significant step that shifts the clinical assessment to other manageable processes, allowing for a more focused and effective management plan to be applied to this subgroup of patients.

The study further found that 23 patients (n=23; 57.5%) had abnormalities on both endoscopy and HRM, which shows that a subset of patients has both structural and functional elements that contribute to their symptoms. However, the fact that some patients have HRM abnormalities in isolation without endoscopic abnormality underlines the importance that NCCP is frequently caused by discrete functional disorders not visible by endoscopic examination. Clinically, this finding emphasizes the role of HRM in diagnostic clarification, particularly in patients with ongoing symptoms in the face of normal endoscopic findings. The predominance of inflammatory mucosal changes (pangastritis, *H. pylori*-related gastritis) also suggests an important role of mucosal irritation in the pathophysiology of NCCP. Together, these findings underscore the multifactorial basis of NCCP and the need for rigorous diagnostic assessment in order to tailor individual treatment strategies.

Comparison with existing literature

Our cohort (high incidence of IEM in around 30% of NCCP patients) is comparable to many other international series. IEM has remained a regular component of the list of the most prevalent motility disorders in Western populations: a multicenter study found IEM with altered esophageal peristalsis in nearly 29.1% of patients [10]. Yet another systematic review has confirmed that IEM is a common motility abnormality captured on HRM [11]. These figures are very similar to those reported by us and further highlight the worldwide distribution of NCCP non-obstructive motility abnormalities. Cohort studies from Asian populations, however, show differences in prevalence. A study from Korea reported esophageal motility disorders in 41% of dysphagia patients, indicating a similarly high burden of functional esophageal pathologies in the region [12]. Our results are consistent with this trend, suggesting a common pathophysiology of the Middle Eastern and Asian populations. With respect to achalasia, its relative rarity is well-established worldwide. Meta-analytic estimates indicate an achalasia prevalence of 0.7-15 per 100,000 persons, which is consistent with the low prevalence of achalasia and outflow obstruction of the EGJ in our cohort (~5-7%) [13-16]. This further supports the fact that major motility disorders are still rare in specialized cohorts. The use of HRM and the Chicago Classification in our study aligns with established methodologies for classifying esophageal motility disorders.

Clinical implications and public health relevance

The results of this study have important implications for clinical practice, especially for the diagnostic evaluation and management of NCCP. First, the high prevalence of motility disorders found only by HRM reminds us of the critical importance of this diagnostic tool. Endoscopic findings are normal in most cases of unexplained chest pain, and clinicians should consider early referral for HRM. Endoscopy alone is often inadequate for the diagnosis of motility disorders, which can cause patients to continue to suffer with symptoms and be treated inappropriately. The application of standardized diagnostic algorithms combining HRM at an early stage may increase diagnostic accuracy and lead to timely, appropriate management and better patient outcomes. From a therapeutic standpoint, the ability to point to specific cause(s) of motility dysfunction can drive specific intervention. Similarly, prokinetic agents, dietary changes, and behavioral therapy to improve esophageal clearance may be helpful in patients with IEM. Patients with DES or achalasia may need more specialized therapies such as botulinum neurotoxin (Botox) injections, pneumatic dilatation, or peroral endoscopic myotomy (POEM). Accurate diagnostic classification is important to avoid the trial-and-error approach that is often seen in the management of NCCP. On a more public health level, the findings point to several possible preventive intervention options. As the prevalence of pangastritis and *H. pylori *positivity is high, community-based screening and *H. pylori *eradication programs might be appropriate to curb the burden of gastric inflammation and associated symptoms. In addition, lifestyle interventions to change dietary practices, decrease obesity, and increase exercise may serve to ameliorate risk factors for both structural lesions like hiatus hernia and functional disorders like IEM.

Limitations and future directions

One of the key points that should be stated in this research is that, although several useful findings were made regarding the prevalence and disorder of esophageal motility among NCCP patients, several limitations can be outlined. In brief, the primary limitation is that only males were included in the study cohort. This makes it difficult to generalize the study findings, as the existence of sex-specific differences in esophageal motor activity and symptom perception is well-reported in the literature. The female subjects used in future studies are necessary to provide a more detailed picture of NCCP in the general population. A retrospective design inherently introduces bias, including the use of existing medical records and potential variability in data collection. Prospective studies on standardized protocols should verify the causal relationships between clinical variables and motility disorders. Because this was a single-center study conducted in a tertiary referral hospital, the patient population may not fully represent the broader community, introducing a potential referral bias that could influence the observed prevalence of motility disorders. The relatively small sample size limits the statistical power of the study, particularly for subgroup analyses and multivariable regression, increasing the risk of type II errors (i.e., failing to identify true associations). The size of the patients included in the study, along with the inclusion of multicenter partnerships, could help address this weakness. The absence of longitudinal follow-up is another limitation that prevents analysis of the treatment outcome and addressing symptom resolution in the long term. Additionally, there was a temporal lag between data collection (2017-2018) and manuscript review (2025). Changes in referral patterns, diagnostic criteria, and population demographics over this period may limit the direct comparability of our findings to current clinical practice. Nevertheless, these data remain valuable as one of the first regional assessments of esophageal motility in NCCP using HRM. Future research should include follow-up evaluation as a means of establishing the effectiveness of some interventions based on the outcomes of HRM. This would provide effective data for clinical decision-making and ultimately lead to improved patient care.

## Conclusions

This study highlights the utility of HRM in diagnosing esophageal motility disorders in patients presenting with NCCP. IEM was the most common abnormality, exhibiting the same distribution pattern as in Western and Asian populations. However, major motility abnormalities, such as achalasia and EGJ outflow obstruction, were relatively uncommon. Notably, the proportion of patients with normal endoscopic images and abnormal HRM outcomes was high, providing evidence that standard diagnostic tools, such as endoscopy alone, do not have sufficient power to detect functional esophageal disorders. The research also presents findings of the Middle East region, where there is a lack of research on esophageal motility patterns. The overrepresentation of male patients in our cohort reflects both local referral patterns and the need for future studies that include women, as well as larger and more diverse cohorts of patients. These results further support the clinical value of adding HRM to diagnostic algorithms for NCCP, particularly for patients in whom the initial cardiac/endoscopic findings are equivocal. In conclusion, this study supports the role of HRM as a key functional diagnostic tool that complements endoscopy in NCCP evaluation, reinforcing the importance of a multimodal approach to characterize esophageal motor disorders and guide targeted management accurately.
